# Supplementary material for: Identifying genetic variants associated with chromatin looping and genome function
Source: Nat Commun. 2024 Sep 18;15:8174. doi: 10.1038/s41467-024-52296-4 (PMC11408621; doi:10.1038/s41467-024-52296-4)
Supplement: Supplementary file 1 — Supplementary Information [file 41467_2024_52296_MOESM1_ESM.pdf]

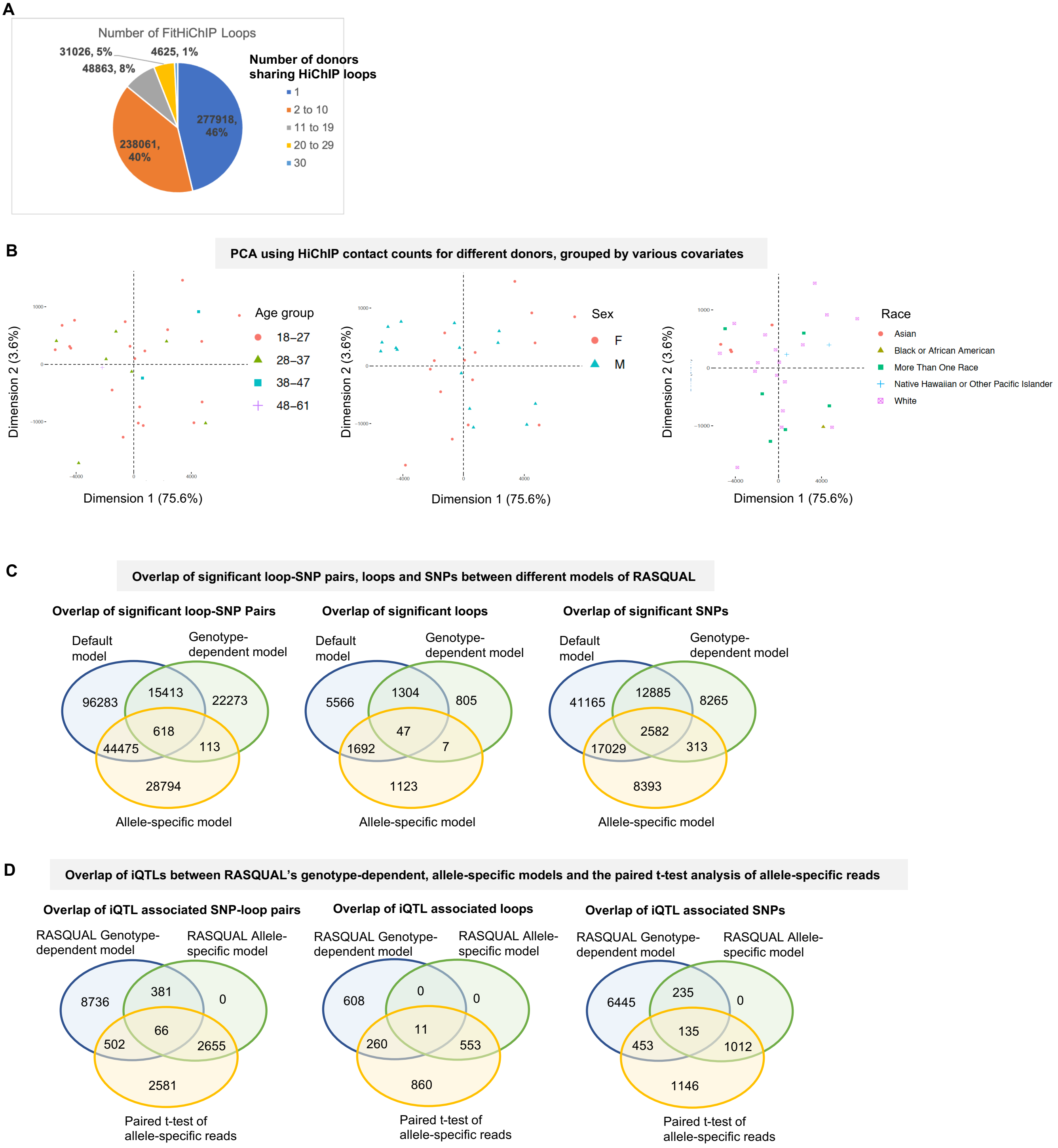
A
Number of FitHiChIP Loops
31026, 5%
4625, 1%
48863, 8%
277918, 46%
238061, 40%
Number of donors sharing HiChIP loops
1
2 to 10
11 to 19
20 to 29
30
B
PCA using HiChIP contact counts for different donors, grouped by various covariates
Dimension 2 (3.6%)
Dimension 1 (75.6%)
Age group
18–27
28–37
38–47
48–61
Sex
F
M
Race
Asian
Black or African American
More Than One Race
Native Hawaiian or Other Pacific Islander
White
C
Overlap of significant loop-SNP pairs, loops and SNPs between different models of RASQUAL
Overlap of significant loop-SNP Pairs
Default model
Genotype-dependent model
Allele-specific model
96283
15413
22273
618
44475
113
28794
Overlap of significant loops
Default model
Genotype-dependent model
Allele-specific model
5566
1304
805
47
1692
7
1123
Overlap of significant SNPs
Default model
Genotype-dependent model
Allele-specific model
41165
12885
8265
2582
17029
313
8393
D
Overlap of iQTLs between RASQUAL's genotype-dependent, allele-specific models and the paired t-test analysis of allele-specific reads
Overlap of iQTL associated SNP-loop pairs
RASQUAL Genotype-dependent model
RASQUAL Allele-specific model
Paired t-test of allele-specific reads
8736
381
0
66
502
2655
2581
Overlap of iQTL associated loops
RASQUAL Genotype-dependent model
RASQUAL Allele-specific model
Paired t-test of allele-specific reads
608
0
0
11
260
553
860
Overlap of iQTL associated SNPs
RASQUAL Genotype-dependent model
RASQUAL Allele-specific model
Paired t-test of allele-specific reads
6445
235
0
135
453
1012
1146

Supplementary Fig. 1: Donor-wise HiChIP loop statistics and comparison between RASQUAL models for iQTL derivation: (A) Count of FitHiChIP loops significant in a specific number of donors. Source data are provided as a Source Data file. **(B)** Principal component analysis (PCA) using HiChIP contact counts of all donors. Donors are categorized by age group, sex, and race. **(C)** Overlap between three different RASQUAL model outputs (FDR 5%), between SNP-loop pairs (left), loops (middle) and SNPs (right). **(D)** For the final set of iQTL associations, overlap of iQTL associated SNP-loop pairs (left), loops (middle) and SNPs (right) according to their significance in RASQUAL genotype-dependent model, allele-specific model, and the paired t-test of the allele-specific reads.

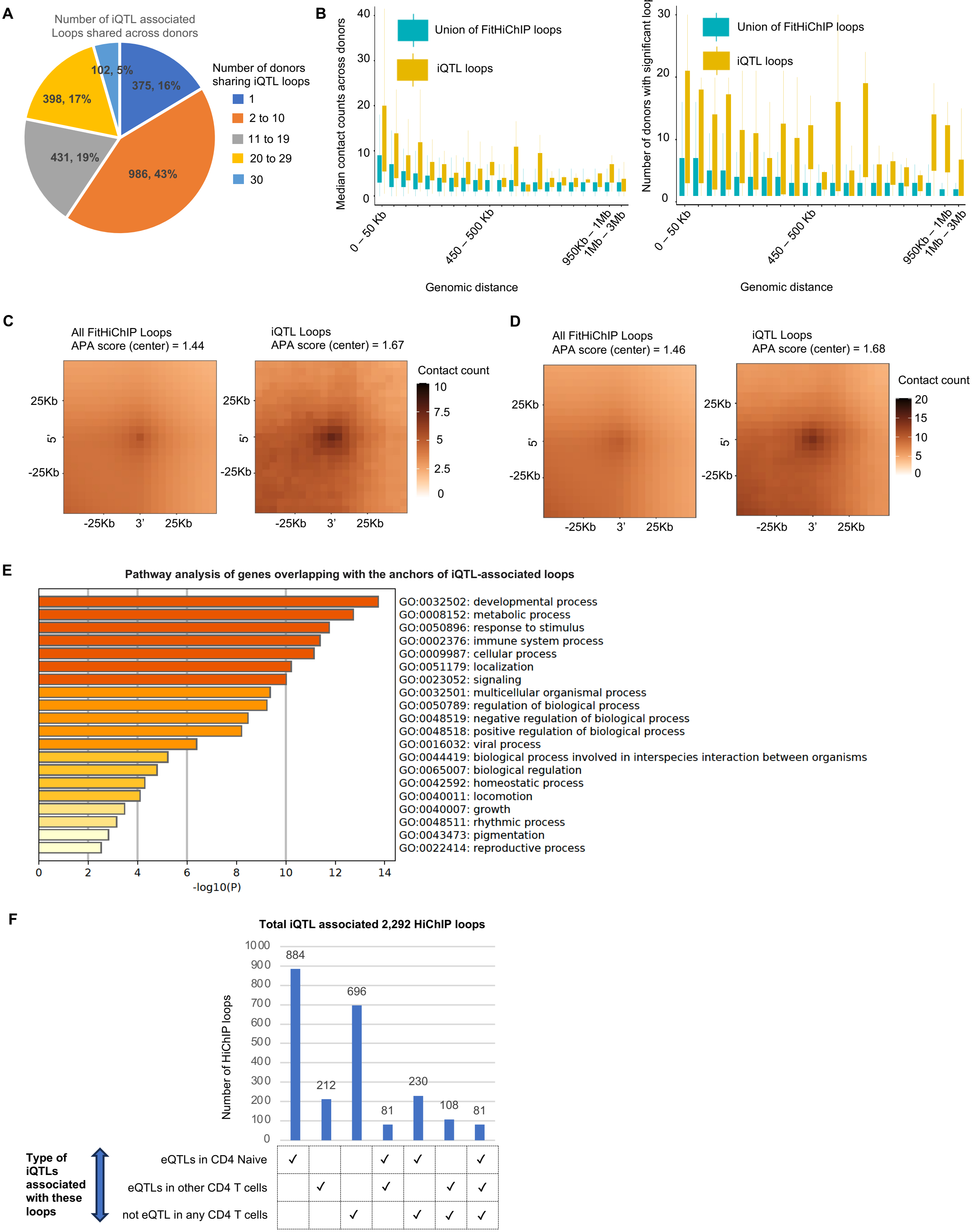

A
Number of iQTL associated Loops shared across donors
375, 16%
986, 43%
431, 19%
398, 17%
102, 5%
Number of donors sharing iQTL loops
1
2 to 10
11 to 19
20 to 29
30
B
Median contact counts across donors
Union of FitHiChIP loops
iQTL loops
0 – 50 Kb
450 – 500 Kb
950Kb – 1Mb
1Mb – 3Mb
Genomic distance
Number of donors with significant loop
Union of FitHiChIP loops
iQTL loops
0 – 50 Kb
450 – 500 Kb
950Kb – 1Mb
1Mb – 3Mb
Genomic distance
C
All FitHiChIP Loops
APA score (center) = 1.44
iQTL Loops
APA score (center) = 1.67
Contact count
25Kb
5'
-25Kb
-25Kb
3'
25Kb
D
All FitHiChIP Loops
APA score (center) = 1.46
iQTL Loops
APA score (center) = 1.68
Contact count
E
Pathway analysis of genes overlapping with the anchors of iQTL-associated loops
GO:0032502: developmental process
GO:0008152: metabolic process
GO:0050896: response to stimulus
GO:0002376: immune system process
GO:0009987: cellular process
GO:0051179: localization
GO:0023052: signaling
GO:0032501: multicellular organismal process
GO:0050789: regulation of biological process
GO:0048519: negative regulation of biological process
GO:0048518: positive regulation of biological process
GO:0016032: viral process
GO:0044419: biological process involved in interspecies interaction between organisms
GO:0065007: biological regulation
GO:0042592: homeostatic process
GO:0040011: locomotion
GO:0040007: growth
GO:0048511: rhythmic process
GO:0043473: pigmentation
GO:0022414: reproductive process
-log10(P)
F
Total iQTL associated 2,292 HiChIP loops
Number of HiChIP loops
884
212
696
81
230
108
81
Type of iQTLs associated with these loops
eQTLs in CD4 Naive
eQTLs in other CD4 T cells
not eQTL in any CD4 T cells

Supplementary Fig. 2: Characteristics of iQTL associated loops: (A) Sharing of iQTL-associated loops among different numbers of donors. **(B)** Comparison between the complete set of FitHiChIP loops used as an input to iQTL analysis, and the loops associated with iQTLs: median contact counts across all donors (left), number of donors having these loops as significant (right). Boxplots indicate 25th, 75th percentiles (box). **(C)** Aggregate peak analysis (APA) for the complete set of FitHiChIP loops (left) and the loops associated with iQTLs (right). APA score indicates higher enrichment of the given set of loops compared to the background set of complete HiChIP contacts. **(D)** Similar APA with respect to the merged CD4 T cell Hi-C data provided in Shi et. al. medRxiv 2023 paper. **(E)** Pathway analysis for the genes overlapping with the anchors (interacting bins) of the iQTL associated loops. Metascape was used for the analysis with default input parameters and top pathways with FDR < 5% were reported. **(F)** Number of HiChIP loops according to their associated iQTLs: whether the constituent iQTLs of a loop are eQTLs in CD4 Naïve T cells, eQTLs in other CD4 T cell subsets, not eQTL in any CD4 T cells, or a combination of these three categories (i.e., multiple iQTLs with different overlap features). For example, there are 230 HiChIP loops such that they are associated with at least one iQTL which is an eQTL in Naive CD4 T cell, and at least one iQTL which is not an eQTL in any CD4 T cells. APA: aggregate peak analysis, GO: gene ontology.

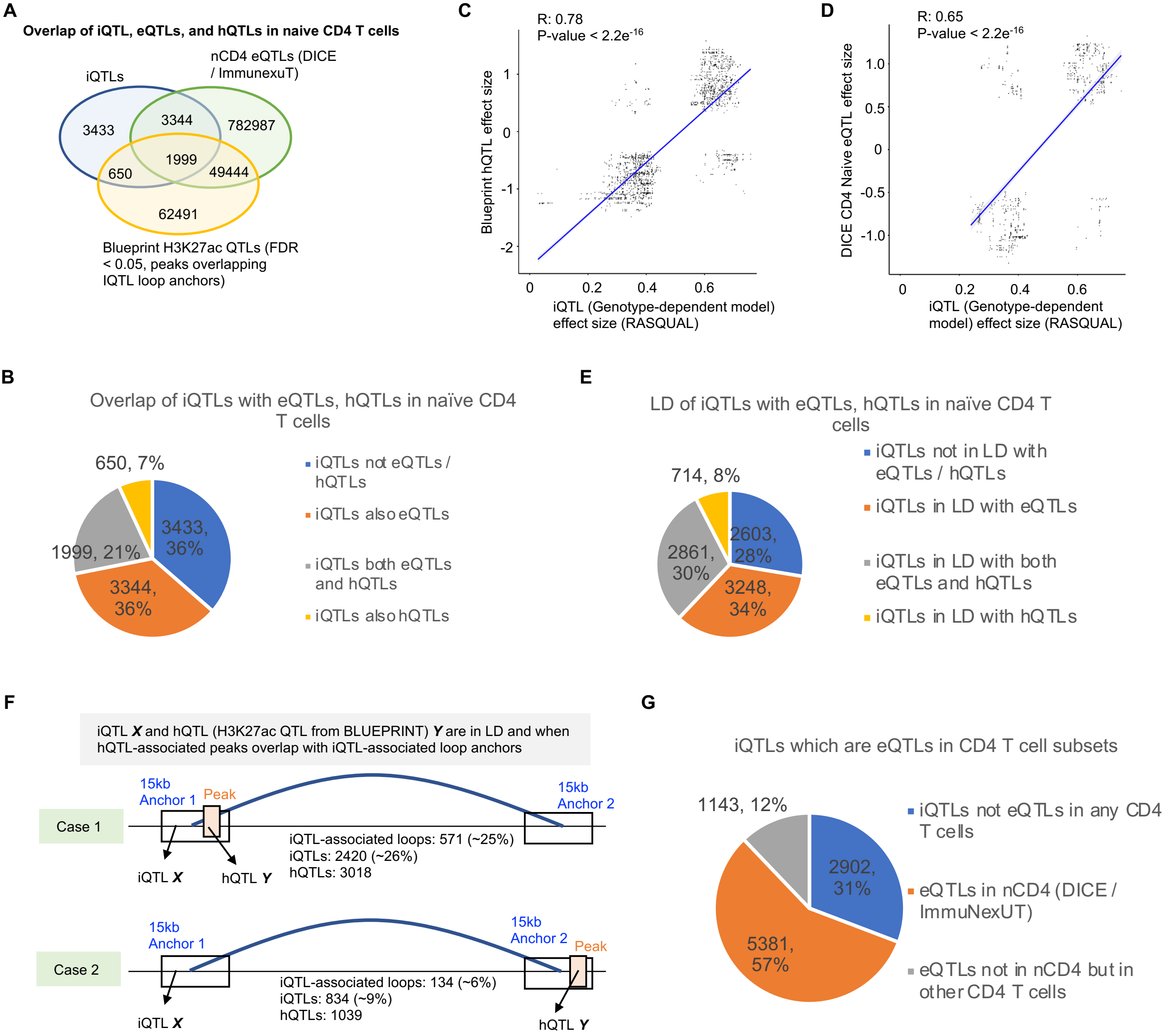
A
Overlap of iQTL, eQTLs, and hQTLs in naive CD4 T cells
iQTLs
nCD4 eQTLs (DICE / ImmunexuT)
3433
3344
782987
1999
650
49444
62491
Blueprint H3K27ac QTLs (FDR < 0.05, peaks overlapping IQTL loop anchors)
C
R: 0.78
P-value < 2.2e-16
Blueprint hQTL effect size
iQTL (Genotype-dependent model) effect size (RASQUAL)
D
R: 0.65
P-value < 2.2e-16
DICE CD4 Naive eQTL effect size
iQTL (Genotype-dependent model) effect size (RASQUAL)
B
Overlap of iQTLs with eQTLs, hQTLs in naïve CD4 T cells
650, 7%
1999, 21%
3433, 36%
3344, 36%
iQTLs not eQTLs / hQTLs
iQTLs also eQTLs
iQTLs both eQTLs and hQTLs
iQTLs also hQTLs
E
LD of iQTLs with eQTLs, hQTLs in naïve CD4 T cells
714, 8%
2861, 30%
2603, 28%
3248, 34%
iQTLs not in LD with eQTLs / hQTLs
iQTLs in LD with eQTLs
iQTLs in LD with both eQTLs and hQTLs
iQTLs in LD with hQTLs
F
iQTL X and hQTL (H3K27ac QTL from BLUEPRINT) Y are in LD and when hQTL-associated peaks overlap with iQTL-associated loop anchors
Case 1
15kb Anchor 1
Peak
15kb Anchor 2
iQTL X
hQTL Y
iQTL-associated loops: 571 (~25%)
iQTLs: 2420 (~26%)
hQTLs: 3018
Case 2
15kb Anchor 1
15kb Anchor 2
Peak
iQTL X
hQTL Y
iQTL-associated loops: 134 (~6%)
iQTLs: 834 (~9%)
hQTLs: 1039
G
iQTLs which are eQTLs in CD4 T cell subsets
1143, 12%
2902, 31%
5381, 57%
iQTLs not eQTLs in any CD4 T cells
eQTLs in nCD4 (DICE / ImmuNexUT)
eQTLs not in nCD4 but in other CD4 T cells

Supplementary Fig. 3: Overlap and concordance among iQTLs, eQTLs and histone QTLs: **(A)** Overlap between iQTLs (this study), Blueprint H3K27ac histone QTLs (hQTLs) and nCD4 eQTLs (DICE plus ImmuNexUT). Only the hQTLs with FDR < 0.05 and their associated peaks overlapping with an iQTL loop anchor are considered. **(B)** Similar to (A) where the numbers and percentages of iQTLs overlapping with nCD4 eQTLs (DICE or ImmuNexUT) and the Blueprint H3K27ac QTLs are indicated. **(C)** Correlation of effect sizes for common SNPs between iQTLs (significant by the genotype-dependent model of RASQUAL) and Blueprint H3K27ac hQTLs, provided their associated peaks also overlap with the iQTL loop anchors. Source data are provided as a Source Data file. **(D)** Correlation of effect sizes for common SNPs between iQTLs and DICE nCD4 eQTLs, provided that the TSS of respective eGenes are within 5kb of the iQTL loop anchors. Source data are provided as a Source Data file. **(E)** Numbers and percentages of iQTLs having strong (R^2 > 0.8) LD with either the nCD4 eQTLs (DICE or ImmuNexUT) or the Blueprint H3K27ac QTLs. **(F)** Schematic diagram of strong (R^2 > 0.8) LD between an iQTL *X* and a Blueprint H3K27ac QTL (hQTL) *Y* when the hQTLs and their associated peaks both overlap with one of the loop anchors associated with *X*. *Case 1:* When the hQTL *Y* and its associated peak share the same loop anchor as *X*. *Case 2:* When the hQTL *Y* and its associated peak belong to the other anchor of the loop associated with *X*. The numbers of iQTLs, hQTLs and the iQTL associated loops are also indicated. **(G)** Overlap of iQTL SNPs with reference eQTLs (in either DICE or ImmuNexUT) for various CD4 T cell subsets (including nCD4 and other derived CD4 T cells).

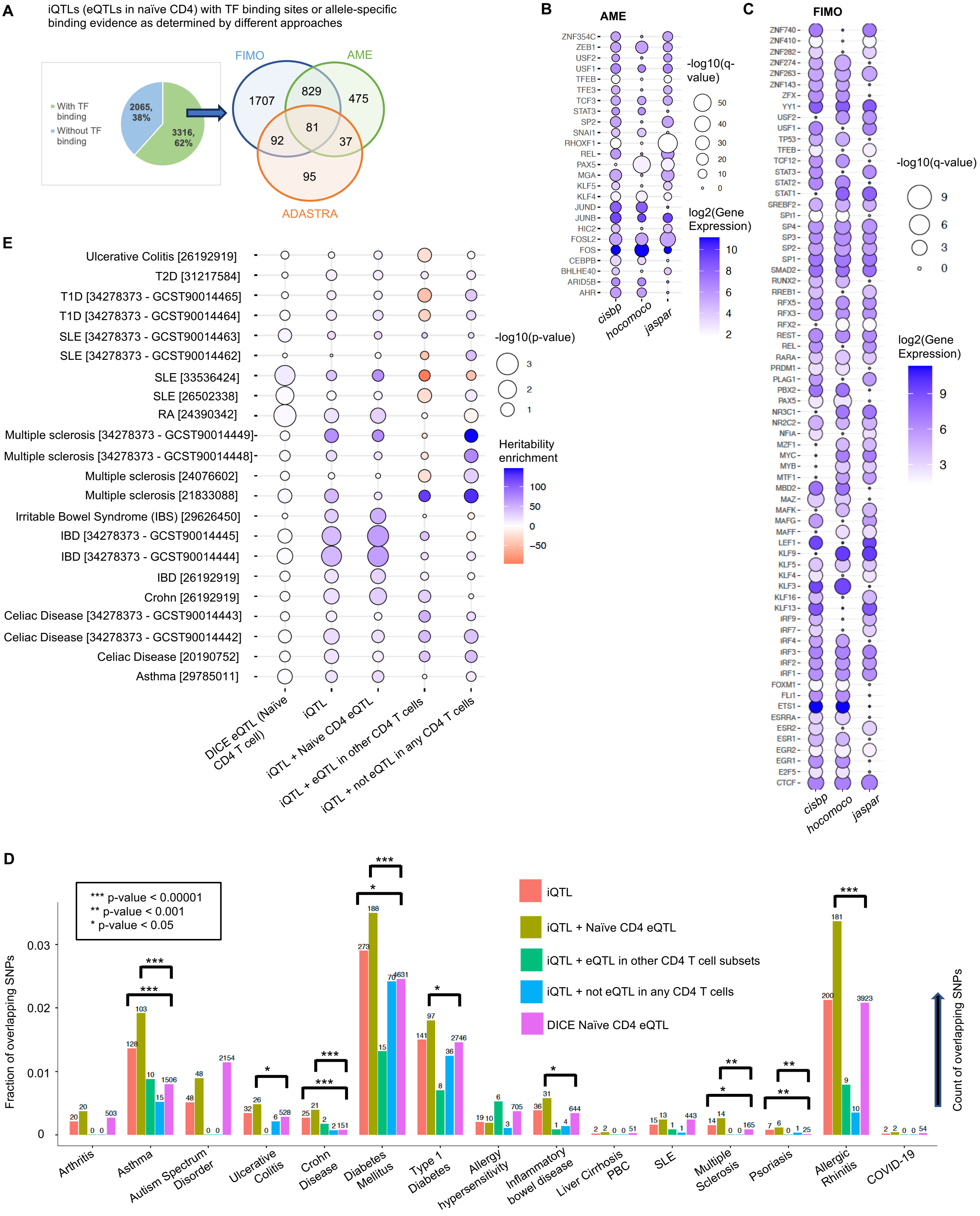
A
iQTLs (eQTLs in naïve CD4) with TF binding sites or allele-specific binding evidence as determined by different approaches
With TF binding
Without TF binding
2065, 38%
3316, 62%
FIMO
AME
ADASTRA
1707
829
475
81
92
37
95
B
AME
ZNF354C
ZEB1
USF2
USF1
TFEB
TFE3
TCF3
STAT3
SP2
SNAI1
RHOXF1
REL
PAX5
MGA
KLF5
KLF4
JUND
JUNB
HIC2
FOSL2
FOS
CEBPB
BHLHE40
ARID5B
AHR
cisbp
hocomoco
jaspar
-log10(q-value)
50
40
30
20
10
0
log2(Gene Expression)
10
8
6
4
2
C
FIMO
ZNF740
ZNF410
ZNF282
ZNF274
ZNF263
ZNF143
ZFX
YY1
USF2
USF1
TP53
TFEB
TCF12
STAT3
STAT2
STAT1
SREBF2
SPI1
SP4
SP3
SP2
SP1
SMAD2
RUNX2
RREB1
RFX5
RFX3
RFX2
REST
REL
RARA
PRDM1
PLAG1
PBX2
PAX5
NR3C1
NR2C2
NFIA
MZF1
MYC
MYB
MTF1
MBD2
MAZ
MAFK
MAFG
MAFF
LEF1
KLF9
KLF5
KLF4
KLF3
KLF16
KLF13
IRF9
IRF7
IRF4
IRF3
IRF2
IRF1
FOXM1
FLI1
ETS1
ESRRA
ESR2
ESR1
EGR2
EGR1
E2F5
CTCF
cisbp
hocomoco
jaspar
-log10(q-value)
9
6
3
0
log2(Gene Expression)
9
6
3
E
Ulcerative Colitis [26192919]
T2D [31217584]
T1D [34278373 - GCST90014465]
T1D [34278373 - GCST90014464]
SLE [34278373 - GCST90014463]
SLE [34278373 - GCST90014462]
SLE [33536424]
SLE [26502338]
RA [24390342]
Multiple sclerosis [34278373 - GCST90014449]
Multiple sclerosis [34278373 - GCST90014448]
Multiple sclerosis [24076602]
Multiple sclerosis [21833088]
Irritable Bowel Syndrome (IBS) [29626450]
IBD [34278373 - GCST90014445]
IBD [34278373 - GCST90014444]
IBD [26192919]
Crohn [26192919]
Celiac Disease [34278373 - GCST90014443]
Celiac Disease [34278373 - GCST90014442]
Celiac Disease [20190752]
Asthma [29785011]
DICE eQTL (Naïve CD4 T cell)
iQTL
iQTL + Naive CD4 eQTL
iQTL + eQTL in other CD4 T cells
iQTL + not eQTL in any CD4 T cells
-log10(p-value)
3
2
1
Heritability enrichment
100
50
0
−50
D
*** p-value < 0.00001
** p-value < 0.001
* p-value < 0.05
iQTL
iQTL + Naïve CD4 eQTL
iQTL + eQTL in other CD4 T cell subsets
iQTL + not eQTL in any CD4 T cells
DICE Naïve CD4 eQTL
Fraction of overlapping SNPs
Count of overlapping SNPs
0.03
0.02
0.01
0
Arthritis
Asthma
Autism Spectrum Disorder
Ulcerative Colitis
Crohn Disease
Diabetes Mellitus
Type 1 Diabetes
Allergy hypersensitivity
Inflammatory bowel disease
Liver Cirrhosis PBC
SLE
Multiple Sclerosis
Psoriasis
Allergic Rhinitis
COVID-19

Supplementary Fig. 4: Characterization of iQTLs which are also eQTLs in naïve CD4 T cells: **(A)** iQTLs overlapping nCD4 eQTLs with or without TF binding motifs (left); overlap of such iQTLs with TF binding motifs from different approaches – *de novo* motif analysis using FIMO, allele-specific motif enrichment by AME, and ADASTRA TF database. For AME and FIMO, iQTLs showing TF binding motifs with respect to any of the three databases cisbp, hocomoco or JASPAR are considered. **(B-C)** TF motif enrichment by AME (B) and FIMO (C) for these iQTLs. Motifs significant in at least two of the three motif databases (cisbp, hocomoco or JASPAR) are shown. TFs with gene expression > 1 TPM having motifs with p-value < 1e-6 are plotted. Source data are provided as a Source Data file. **(D)** Overlap of different categories of iQTLs and reference nCD4 eQTLs (from the DICE database) with the fine-mapped GWAS SNPs for various immune diseases provided in the CausalDB database. Here, p-values are computed using Fisher's exact test. Numbers on top of individual bars indicate the count of overlapping SNPs. **(E)** Heritability enrichment computed by stratified LD score regression (S-LDSC) for different categories of iQTLs and nCD4 DICE eQTLs. GWAS SNPs from various immune diseases are used for analyzing heritability. For individual studies, PubMed IDs and dataset IDs are mentioned within square brackets.

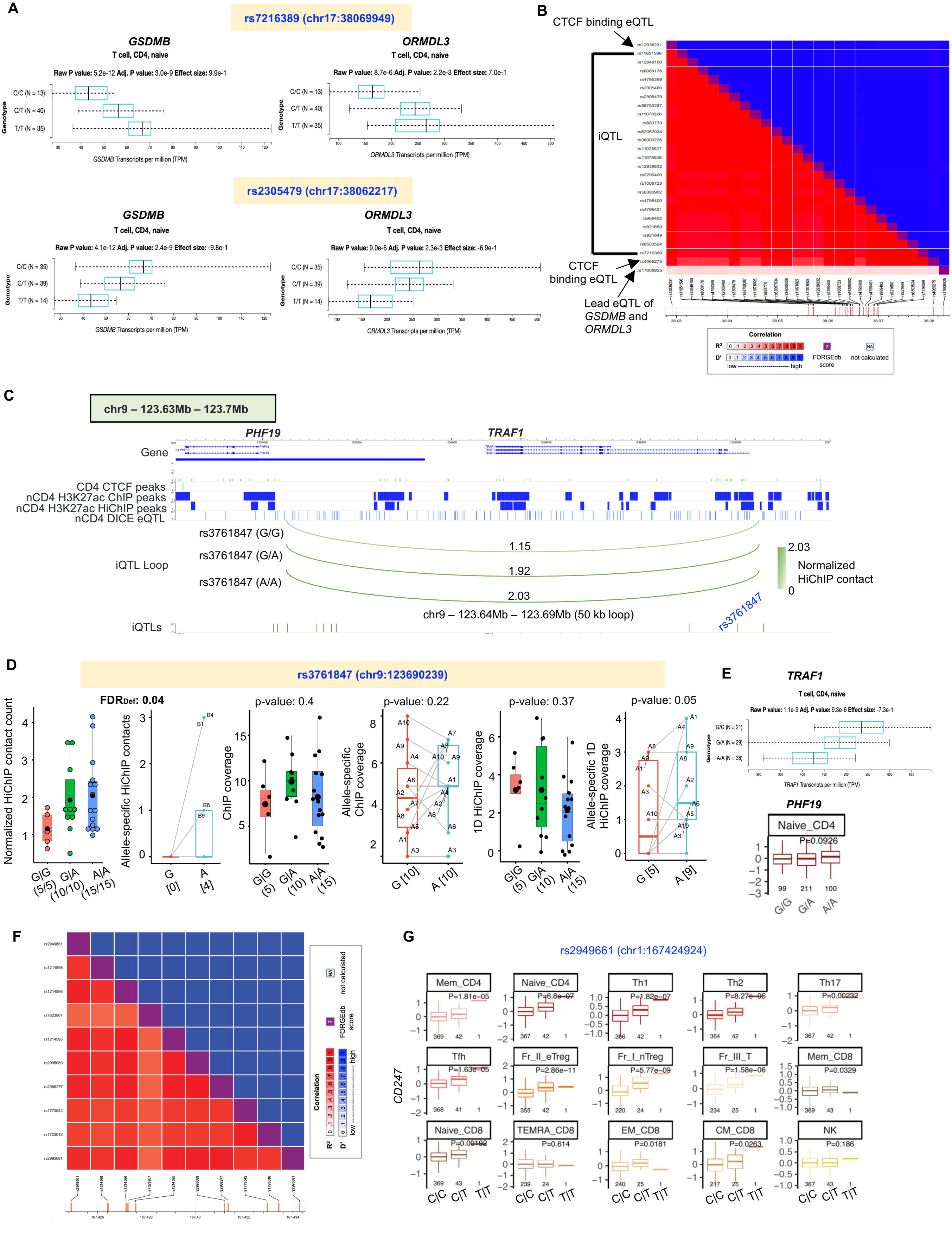
A
rs7216389 (chr17:38069949)
GSDMB
T cell, CD4, naive
Raw P value: 5.2e-12 Adj. P value: 3.0e-9 Effect size: 9.9e-1
ORMDL3
T cell, CD4, naive
Raw P value: 8.7e-6 Adj. P value: 2.2e-3 Effect size: 7.0e-1
C/C (N = 13)
C/T (N = 40)
T/T (N = 35)
Genotype
GSDMB Transcripts per million (TPM)
ORMDL3 Transcripts per million (TPM)
rs2305479 (chr17:38062217)
GSDMB
T cell, CD4, naive
Raw P value: 4.1e-12 Adj. P value: 2.4e-9 Effect size: -9.8e-1
ORMDL3
T cell, CD4, naive
Raw P value: 9.0e-6 Adj. P value: 2.3e-3 Effect size: -6.9e-1
C/C (N = 35)
C/T (N = 39)
T/T (N = 14)
B
CTCF binding eQTL
iQTL
CTCF binding eQTL
Lead eQTL of GSDMB and ORMDL3
Correlation
R²
D'
low high
FORGEdb score
not calculated
C
chr9 – 123.63Mb – 123.7Mb
PHF19
TRAF1
Gene
CD4 CTCF peaks
nCD4 H3K27ac ChIP peaks
nCD4 H3K27ac HiChIP peaks
nCD4 DICE eQTL
rs3761847 (G/G)
rs3761847 (G/A)
rs3761847 (A/A)
iQTL Loop
1.15
1.92
2.03
2.03
Normalized HiChIP contact
0
chr9 – 123.64Mb – 123.69Mb (50 kb loop)
rs3761847
iQTLs
D
rs3761847 (chr9:123690239)
FDRDef: 0.04
p-value: 0.4
p-value: 0.22
p-value: 0.37
p-value: 0.05
Normalized HiChIP contact count
Allele-specific HiChIP contacts
ChIP coverage
Allele-specific ChIP coverage
1D HiChIP coverage
Allele-specific 1D HiChIP coverage
G|G (5/5) G|A (10/10) A|A (15/15)
G [0] A [4]
G|G (5) G|A (10) A|A (15)
G [10] A [10]
G [5] A [9]
E
TRAF1
T cell, CD4, naive
Raw P value: 1.1e-9 Adj. P value: 9.3e-6 Effect size: -7.3e-1
G/G (N = 21)
G/A (N = 29)
A/A (N = 38)
TRAF1 Transcripts per million (TPM)
PHF19
Naive_CD4
P=0.0926
99 211 100
G|G G|A A|A
F
Correlation
R²
D'
low high
FORGEdb score
not calculated
G
rs2949661 (chr1:167424924)
CD247
Mem_CD4 P=1.81e-05
Naive_CD4 P=6.8e-07
Th1 P=1.82e-07
Th2 P=8.27e-05
Th17 P=0.00232
Tfh P=1.63e-05
Fr_II_eTreg P=2.86e-11
Fr_I_nTreg P=5.77e-09
Fr_III_T P=1.58e-06
Mem_CD8 P=0.0329
Naive_CD8 P=0.00192
TEMRA_CD8 P=0.614
EM_CD8 P=0.0181
CM_CD8 P=0.0263
NK P=0.186
C|C C|T T|T

Supplementary Fig. 5: Examples of iQTLs which are also eQTLs in CD4 Naïve T cell: (A) Genotype-dependent variation of gene expression for two iQTLs rs7216389 (top) and rs2305479 (bottom) for the genes *ORMDL3* and *GSDMB*, with respect to the DICE nCD4 eQTLs. Boxplots show median (middle lines), 25th, 75th percentiles (box) and minimum and maximum range (whiskers). **(B)** Linkage Disequilibrium (LD) between iQTLs associated with the 40kb HiChIP loop in *ORMDL3* locus (**Fig. 2A**), two CTCF binding eQTLs (described in Schmiedel et al. Nature Communications 2016) and the lead eQTL (indicated) of the genes *GSDMB* and *ORMDL3*. **(C)** Example of an iQTL rs3761847 associated with a 50kb loop in the *TRAF1* locus. Color scale of the green arcs and the values indicate mean normalized HiChIP contact counts by genotype for this SNP. **(D)** Trends of genotype-dependent sequencing depth normalized HiChIP contact counts, ChIP-seq coverage, 1D HiChIP coverage, and allele-specific variation of HiChIP contacts, ChIP and 1D HiChIP reads, for the iQTL rs3761847 with respect to this 50kb HiChIP loop. For genotype dependent trends, X axis indicates different SNP genotypes, numbers in the formats (***a*** / ***b***) or (***b***) denote that the corresponding genotype is present in ***b*** donors, out of which ***a*** donors have this HiChIP loop as significant (by FitHiChIP). For allele-specific trends, X axis denotes different alleles, and numbers in the format [*c*] indicate that *c* heterozygous donors have nonzero reads for this allele. FDR_{Def} denotes the statistical significance (FDR) of this iQTL SNP-loop pair using the default RASQUAL model. For genotype-specific plots, p-values are obtained from linear regression (ANOVA) while for allele-specific plots, p-values are computed by one-sided paired t-test. Boxplots show median (middle lines), mean (bigger black dots), 25th, 75th percentiles (box) and individual samples (smaller black dots or dots with symbols A* where * indicates numbers). **(E)** Genotype dependent variation of gene expression for the iQTL rs3761847 SNP for the genes *TRAF1* and *PHF19* (adapted from DICE and ImmuNexUT databases, respectively). Boxplots show median (middle lines), 25th, 75th percentiles (box) and minimum and maximum range (whiskers). **(F)** LD between all 10 iQTLs associated with the 40kb HiChIP loop in *CD247* locus depicted in Fig. 2C. **(G)** Genotype dependent variation of *CD247* expression for the iQTL rs2949661 (adapted from the ImmuNexUT database). Boxplots show median (middle lines), 25th, 75th percentiles (box) and minimum and maximum range (whiskers).

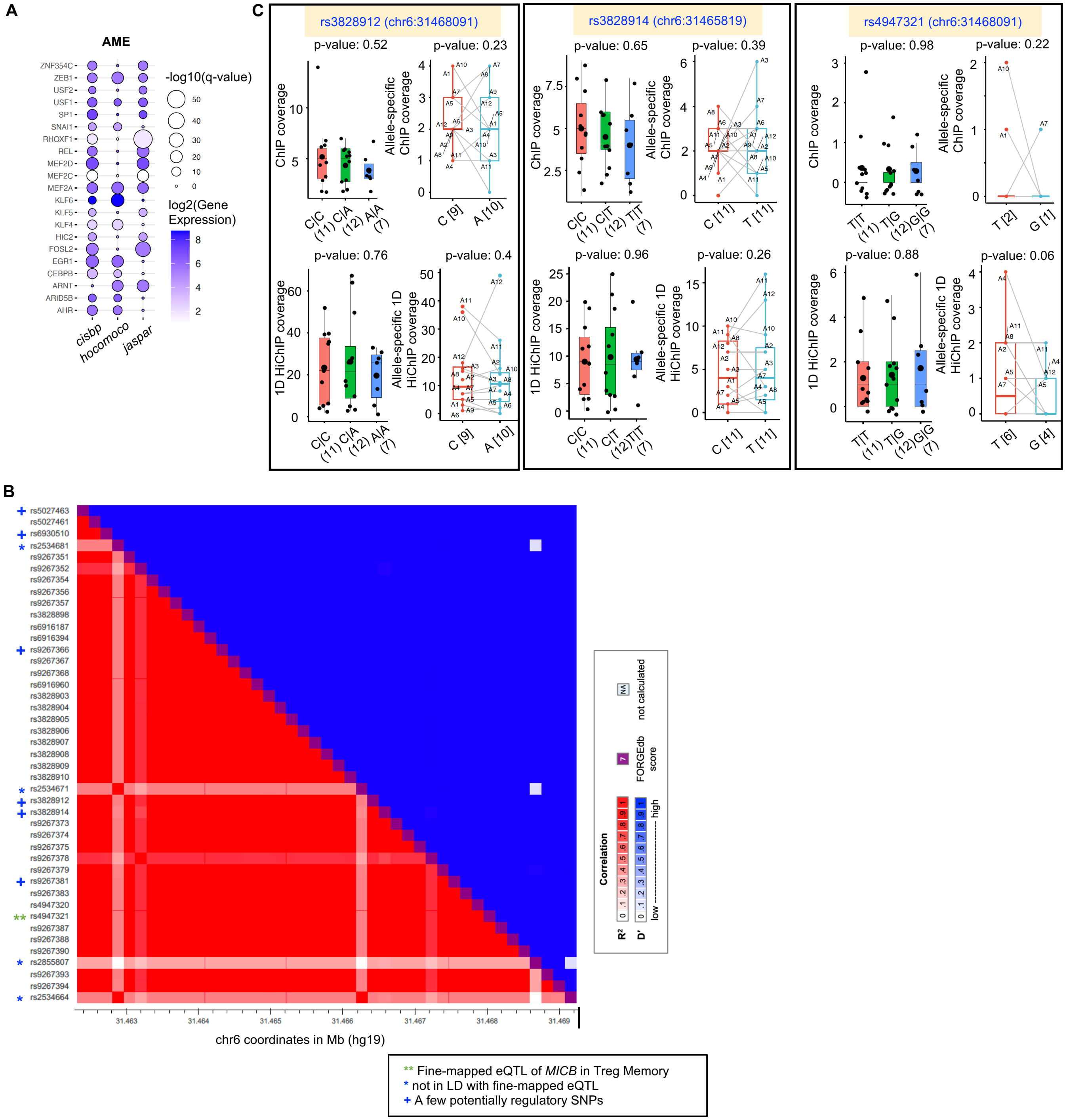

A
AME
ZNF354C
ZEB1
USF2
USF1
SP1
SNAI1
RHOXF1
REL
MEF2D
MEF2C
MEF2A
KLF6
KLF5
KLF4
HIC2
FOSL2
EGR1
CEBPB
ARNT
ARID5B
AHR
cisbp
hocomoco
jaspar
-log10(q-value)
50
40
30
20
10
0
log2(Gene Expression)
8
6
4
2
C
rs3828912 (chr6:31468091)
p-value: 0.52
p-value: 0.23
ChIP coverage
Allele-specific ChIP coverage
C|C (11)
C|A (12)
A|A (7)
C [9]
A [10]
p-value: 0.76
p-value: 0.4
1D HiChIP coverage
Allele-specific 1D HiChIP coverage
rs3828914 (chr6:31465819)
p-value: 0.65
p-value: 0.39
C|C (11)
C|T (12)
T|T (7)
C [11]
T [11]
p-value: 0.96
p-value: 0.26
rs4947321 (chr6:31468091)
p-value: 0.98
p-value: 0.22
T|T (11)
T|G (12)
G|G (7)
T [2]
G [1]
p-value: 0.88
p-value: 0.06
T [6]
G [4]
B
rs5027463
rs5027461
rs6930510
rs2534681
rs9267351
rs9267352
rs9267354
rs9267356
rs9267357
rs3828898
rs6916187
rs6916394
rs9267366
rs9267367
rs9267368
rs6916960
rs3828903
rs3828904
rs3828905
rs3828906
rs3828907
rs3828908
rs3828909
rs3828910
rs2534671
rs3828912
rs3828914
rs9267373
rs9267374
rs9267375
rs9267378
rs9267379
rs9267381
rs9267383
rs4947320
rs4947321
rs9267387
rs9267388
rs9267390
rs2855807
rs9267393
rs9267394
rs2534664
31.463
31.464
31.465
31.466
31.467
31.468
31.469
chr6 coordinates in Mb (hg19)
NA not calculated
FORGEdb score
Correlation
R²
D'
low
high
** Fine-mapped eQTL of MICB in Treg Memory
* not in LD with fine-mapped eQTL
+ A few potentially regulatory SNPs

Supplementary Fig. 6: Characterization of iQTLs which are not eQTLs in naïve CD4 T cells but are eQTLs in other CD4 T cell subsets: (A) TF motif enrichment by AME for iQTLs supported by at least two motif databases out of cisbp, hocomoco or JASPAR. TFs with gene expression > 1 TPM having motifs with p-value < 1e-6 are plotted. Source data are provided as a Source Data file. **(B)** LD between iQTLs of *MICB* locus (Fig. 3C), which are also eQTLs of *MICB* in Treg Memory and other CD4 T cell subsets. **(C)** Trends of genotype-dependent ChIP-seq coverage, 1D HiChIP coverage, and allele-specific variation of ChIP and 1D HiChIP reads, for various iQTLs associated with the HiChIP loops in *MICB* locus, as depicted in Fig. 3C. For genotype dependent trends, X axis indicates different SNP genotypes, numbers in the format (***b***) denote that the corresponding genotype is present in ***b*** donors. For allele-specific trends, X axis denotes different alleles, and numbers in the format [*c*] indicate that *c* heterozygous donors have nonzero reads for this allele. For genotype-specific plots, p-values are obtained from linear regression (ANOVA) while for allele-specific plots, p-values are computed by one-sided paired t-test. Boxplots show mean (bigger black dots), median (middle lines), 25th, 75th percentiles (box) and individual samples (smaller black dots or dots with symbols A* where * indicates numbers).

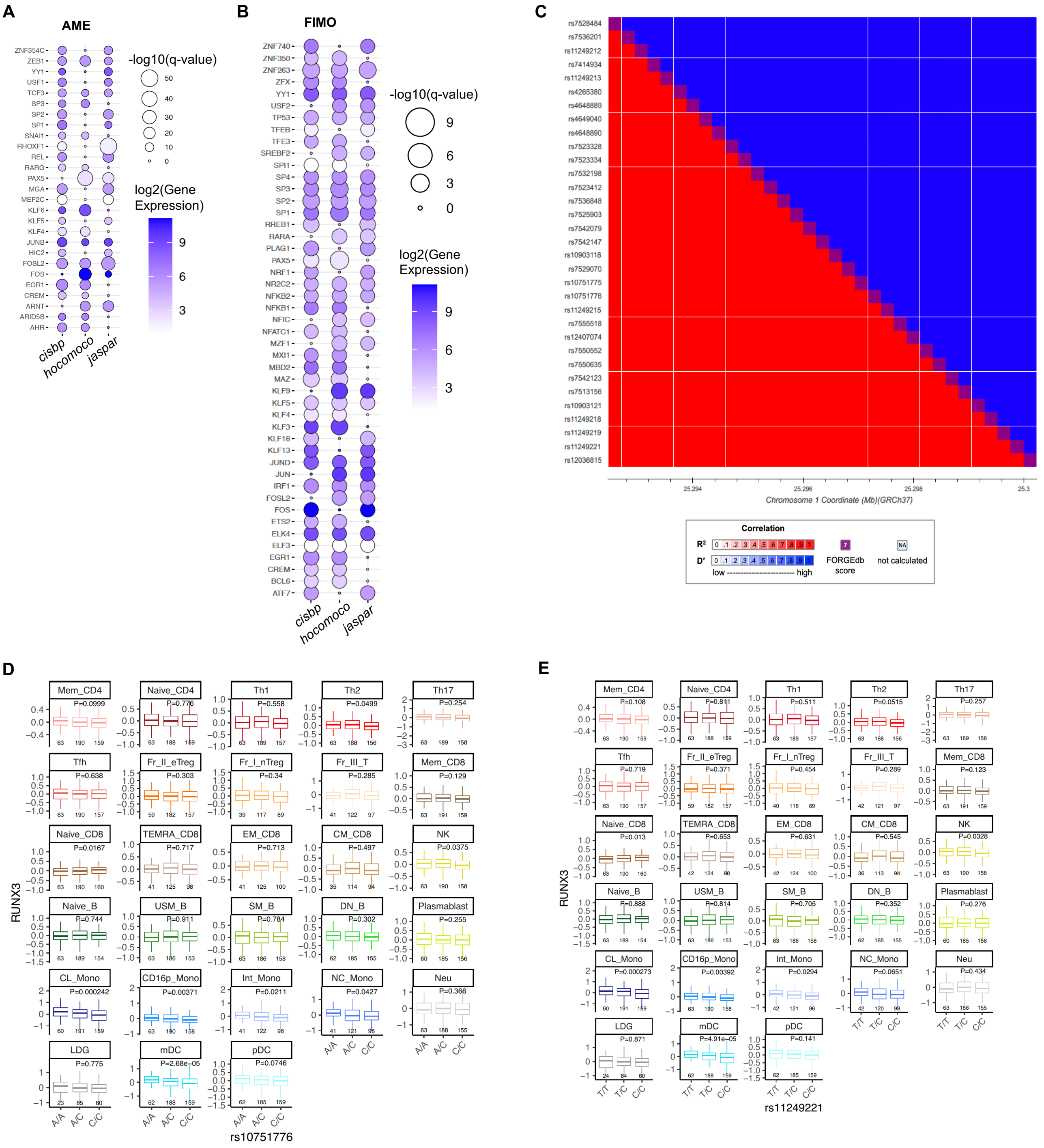

A
AME
ZNF354C
ZEB1
YY1
USF1
TCF3
SP3
SP2
SP1
SNAI1
RHOXF1
REL
RARG
PAX5
MGA
MEF2C
KLF6
KLF5
KLF4
JUNB
HIC2
FOSL2
FOS
EGR1
CREM
ARNT
ARID5B
AHR
cisbp
hocomoco
jaspar
-log10(q-value)
50
40
30
20
10
0
log2(Gene Expression)
9
6
3
B
FIMO
ZNF740
ZNF350
ZNF263
ZFX
YY1
USF2
TP53
TFEB
TFE3
SREBF2
SPI1
SP4
SP3
SP2
SP1
RREB1
RARA
PLAG1
PAX5
NRF1
NR2C2
NFKB2
NFKB1
NFIC
NFATC1
MZF1
MXI1
MBD2
MAZ
KLF9
KLF5
KLF4
KLF3
KLF16
KLF13
JUND
JUN
IRF1
FOSL2
FOS
ETS2
ELK4
ELF3
EGR1
CREM
BCL6
ATF7
cisbp
hocomoco
jaspar
-log10(q-value)
9
6
3
0
log2(Gene Expression)
9
6
3
C
rs7528484
rs7536201
rs11249212
rs7414934
rs11249213
rs4265380
rs4648889
rs4649040
rs4648890
rs7523328
rs7523334
rs7532198
rs7523412
rs7536848
rs7525903
rs7542079
rs7542147
rs10903118
rs7529070
rs10751775
rs10751776
rs11249215
rs7555518
rs12407074
rs7550552
rs7550635
rs7542123
rs7513156
rs10903121
rs11249218
rs11249219
rs11249221
rs12036815
25.294
25.296
25.298
25.3
Chromosome 1 Coordinate (Mb)(GRCh37)
Correlation
R²
D'
low ------------------------ high
FORGEdb score
NA
not calculated
D
RUNX3
Mem_CD4 P=0.0999
Naive_CD4 P=0.776
Th1 P=0.558
Th2 P=0.0499
Th17 P=0.254
Tfh P=0.638
Fr_II_eTreg P=0.303
Fr_I_nTreg P=0.34
Fr_III_T P=0.285
Mem_CD8 P=0.129
Naive_CD8 P=0.0167
TEMRA_CD8 P=0.717
EM_CD8 P=0.713
CM_CD8 P=0.497
NK P=0.0375
Naive_B P=0.744
USM_B P=0.911
SM_B P=0.784
DN_B P=0.302
Plasmablast P=0.255
CL_Mono P=0.000242
CD16p_Mono P=0.00371
Int_Mono P=0.0211
NC_Mono P=0.0427
Neu P=0.366
LDG P=0.775
mDC P=2.68e-05
pDC P=0.0746
A/A
A/C
C/C
rs10751776
E
RUNX3
Mem_CD4 P=0.108
Naive_CD4 P=0.811
Th1 P=0.511
Th2 P=0.0515
Th17 P=0.257
Tfh P=0.719
Fr_II_eTreg P=0.371
Fr_I_nTreg P=0.454
Fr_III_T P=0.289
Mem_CD8 P=0.123
Naive_CD8 P=0.013
TEMRA_CD8 P=0.653
EM_CD8 P=0.631
CM_CD8 P=0.545
NK P=0.0328
Naive_B P=0.888
USM_B P=0.814
SM_B P=0.705
DN_B P=0.352
Plasmablast P=0.276
CL_Mono P=0.000273
CD16p_Mono P=0.00392
Int_Mono P=0.0294
NC_Mono P=0.0651
Neu P=0.434
LDG P=0.871
mDC P=4.91e-05
pDC P=0.141
T/T
T/C
C/C
rs11249221

Supplementary Fig. 7: Characterization of iQTLs which are not eQTLs in any CD4 T cell subset: (A-B) TF motif enrichment by AME (A) and FIMO (B) for iQTLs such that the motifs are significant in at least two of the three motif databases cisbp, hocomoco or JASPAR. TFs with gene expression > 1 TPM having motifs with p-value < 1e-6 are plotted. Source data are provided as a Source Data file. **(C)** Linkage disequilibrium between all 32 iQTLs associated with the 60kb chromatin loop involving the gene *RUNX3*. **(D)** eQTL summary statistics plot of the SNP rs10751776 with respect to the gene *RUNX3* (adapted from ImmuNexUT database). Boxplots show median (middle lines), 25th, 75th percentiles (box) and minimum and maximum range (whiskers). **(E)** Same as (D) for the SNP rs11249221.

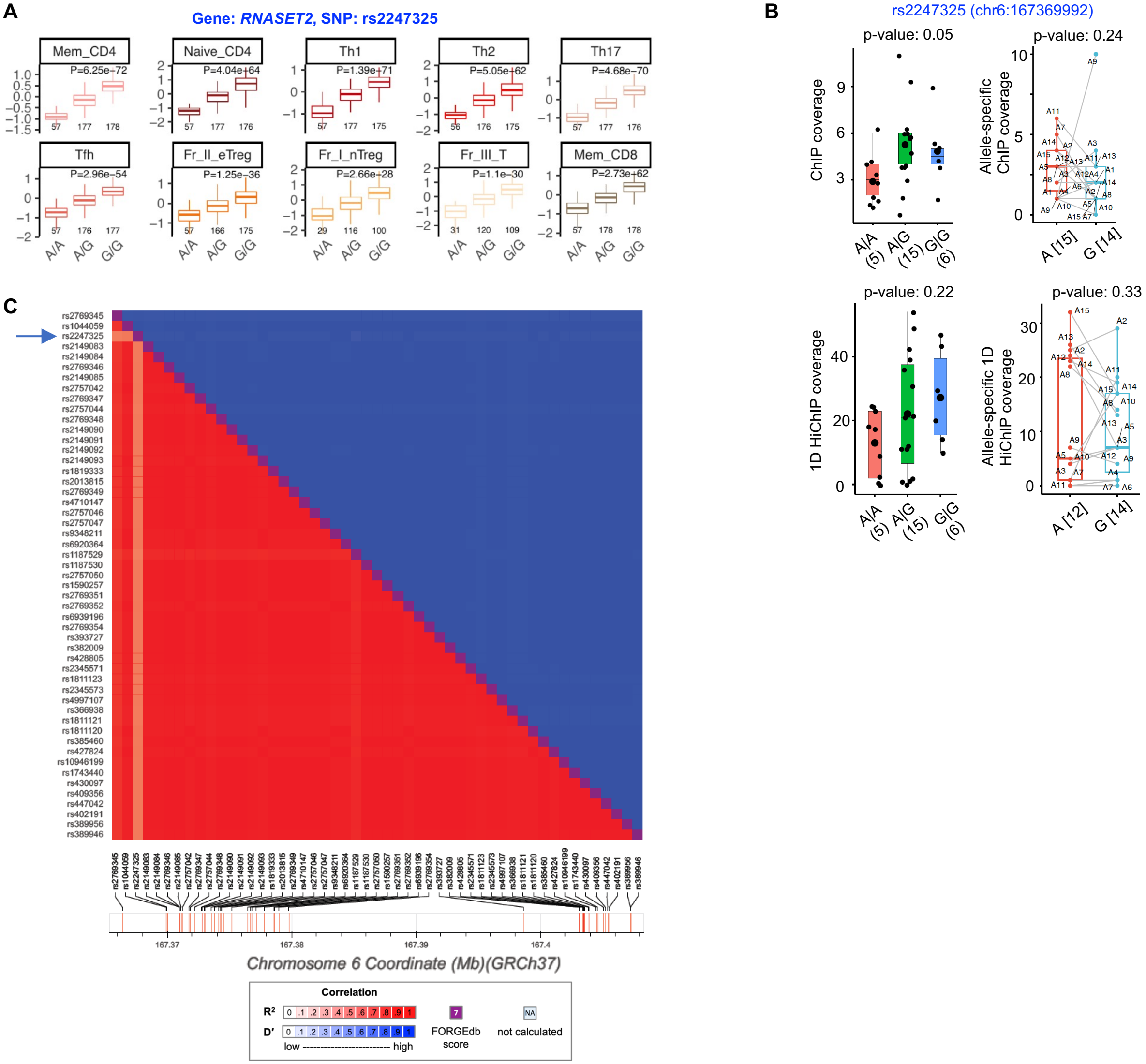
A
Gene: RNASET2, SNP: rs2247325
Mem_CD4
P=6.25e−72
Naive_CD4
P=4.04e−64
Th1
P=1.39e−71
Th2
P=5.05e−62
Th17
P=4.68e−70
Tfh
P=2.96e−54
Fr_II_eTreg
P=1.25e−36
Fr_I_nTreg
P=2.66e−28
Fr_III_T
P=1.1e−30
Mem_CD8
P=2.73e−62
A/A
A/G
G/G
B
rs2247325 (chr6:167369992)
p-value: 0.05
ChIP coverage
p-value: 0.24
Allele-specific ChIP coverage
A [15]
G [14]
p-value: 0.22
1D HiChIP coverage
A/A (5)
A/G (15)
G/G (6)
p-value: 0.33
Allele-specific 1D HiChIP coverage
A [12]
G [14]
C
Chromosome 6 Coordinate (Mb)(GRCh37)
167.37
167.38
167.39
167.4
Correlation
R²
D'
low high
FORGEdb score
not calculated

Supplementary Fig. 8: Properties of the connectivity-QTL rs2247325 and iQTLs in the *RNASET2* locus. (A) eQTL summary statistics plot of the SNP rs2247325 with respect to the gene *RNASET2* (adapted from ImmuNexUT database). Boxplots show median (middle lines), 25th, 75th percentiles (box) and minimum and maximum range (whiskers). **(B)** Trends of genotype-dependent ChIP-seq coverage, 1D HiChIP coverage, and allele-specific variation of ChIP and 1D HiChIP reads, for the connectivity-QTL rs2247325 as depicted in Fig. 6A. For genotype dependent trends, X axis indicates different SNP genotypes, numbers in the format (***b***) denote that the corresponding genotype is present in ***b*** donors. For allele-specific trends, X axis denotes different alleles, and numbers in the format [*c*] indicate that *c* heterozygous donors have nonzero reads for this allele. For genotype-specific plots, p-values are obtained from linear regression (ANOVA) while for allele-specific plots, p-values are computed by one-sided paired t-test. Boxplots show mean (bigger black dots), median (middle lines), 25th, 75th percentiles (box) and individual samples (smaller black dots or dots with symbols A* where * indicates numbers). Various p-values are computed by one-sided t-test. **(C)** LD between all iQTLs associated with at least one of the 4 HiChIP loops indicated within the *RNASET2* locus. The SNP marked with arrow indicates the connectivity QTL rs2247325.

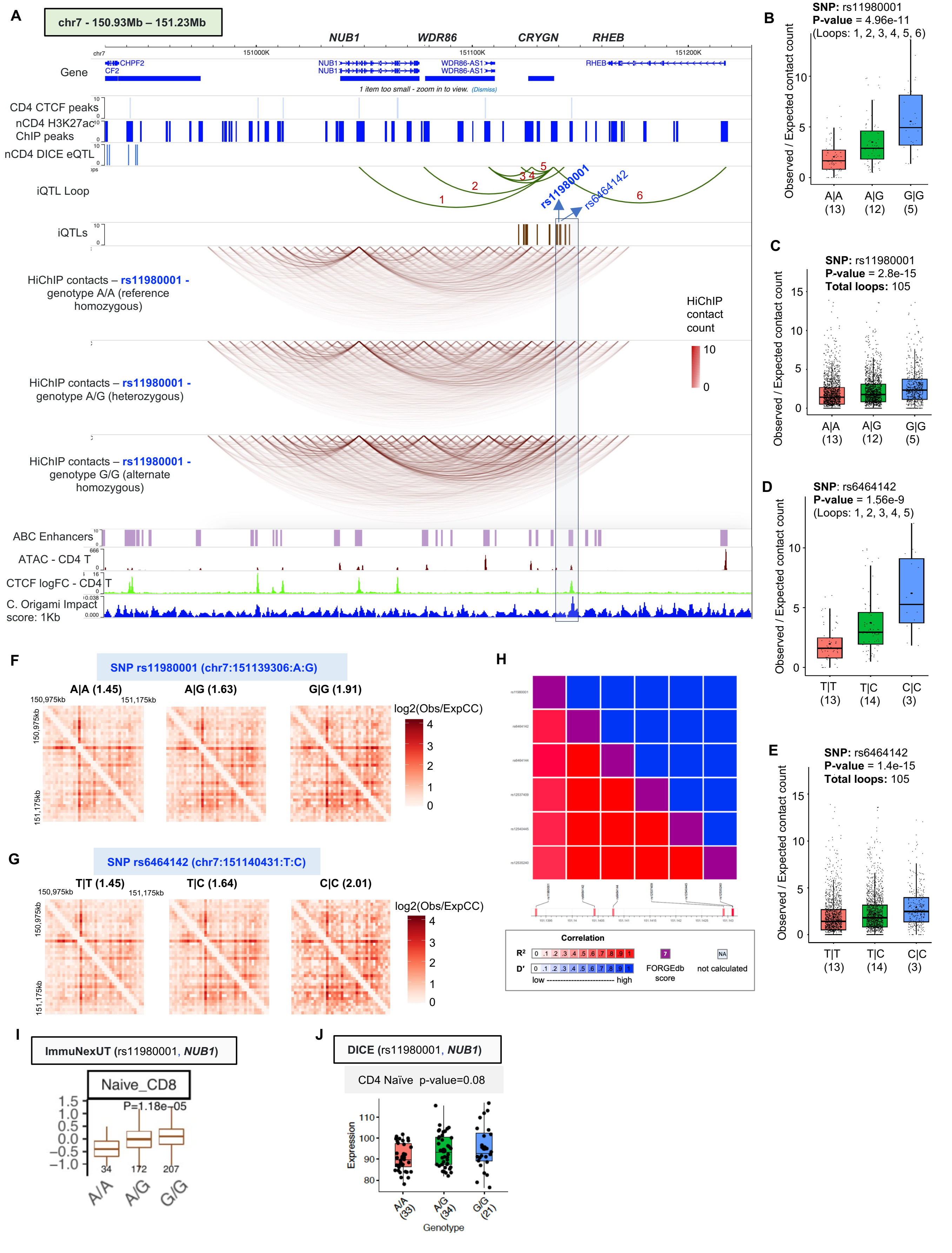

A
chr7 - 150.93Mb – 151.23Mb
NUB1
WDR86
CRYGN
RHEB
Gene
CHPF2
CF2
NUB1
WDR86-AS1
RHEB
1 item too small - zoom in to view. (Dismiss)
CD4 CTCF peaks
nCD4 H3K27ac ChIP peaks
nCD4 DICE eQTL
iQTL Loop
rs11980001
rs6464142
iQTLs
HiChIP contacts – rs11980001 - genotype A/A (reference homozygous)
HiChIP contacts – rs11980001 - genotype A/G (heterozygous)
HiChIP contacts – rs11980001 - genotype G/G (alternate homozygous)
HiChIP contact count
ABC Enhancers
ATAC - CD4 T
CTCF logFC - CD4 T
C. Origami Impact score: 1Kb
B
SNP: rs11980001
P-value = 4.96e-11
(Loops: 1, 2, 3, 4, 5, 6)
Observed / Expected contact count
A|A (13)
A|G (12)
G|G (5)
C
SNP: rs11980001
P-value = 2.8e-15
Total loops: 105
D
SNP: rs6464142
P-value = 1.56e-9
(Loops: 1, 2, 3, 4, 5)
T|T (13)
T|C (14)
C|C (3)
E
SNP: rs6464142
P-value = 1.4e-15
Total loops: 105
F
SNP rs11980001 (chr7:151139306:A:G)
A|A (1.45)
A|G (1.63)
G|G (1.91)
log2(Obs/ExpCC)
G
SNP rs6464142 (chr7:151140431:T:C)
T|T (1.45)
T|C (1.64)
C|C (2.01)
H
Correlation
R²
D'
low ------------------------- high
FORGEdb score
not calculated
I
ImmuNexUT (rs11980001, NUB1)
Naive_CD8
P=1.18e−05
A/A
A/G
G/G
J
DICE (rs11980001, NUB1)
CD4 Naïve p-value=0.08
Expression
Genotype
A/A (33)
A/G (34)
G/G (21)

Supplementary Fig. 9: Example of connectivity-QTLs in the *NUB1* locus. (A) Example of two connectivity-QTLs rs11980001 and rs6464142 associated with 6 and 5 HiChIP loops, respectively, in the *NUB1 l*ocus. Three tracks below iQTLs indicate the changes in overall HiChIP connectivity (color scales indicate the contact counts) according to the genotypes of rs11980001. ABC model enhancers, corresponding to the E2G links of the activated naïve CD4 T cell type, are also shown. Bottom three tracks show CD4 T cell ATAC-seq, CTCF log fold change over control ChIP-seq track (used as an input to C. Origami) and the impact score at 1kb resolution obtained by C. Origami, where higher impact score indicates higher impact on 3D chromatin organization by perturbation of the corresponding loci. **(B)** Genotype-dependent variation of observed / expected contact counts for the SNP rs11980001 and six associated HiChIP loops. The p-value is obtained from linear regression coefficients (ANOVA). Here, X axis denotes different SNP genotypes, and the underlying numbers indicate the corresponding number of donors. Boxplots show median (middle lines), 25th, 75th percentiles (box) and individual samples (black dots). **(C)** Genotype-dependent variation of observed / expected contact counts for the SNP rs11980001 and the complete set of HiChIP loops within *NUB1* locus (as shown in (A)). X axis indicates different genotypes and corresponding number of donors. Boxplots show median (middle lines), 25th, 75th percentiles (box) and individual samples (black dots). The p-value is obtained from linear regression coefficients (ANOVA). **(D)** Same as (B) for the SNP rs6464142. **(E)** Same as (C) for the SNP rs6464142. **(F)** Aggregated contact maps of log2-transformed observed vs expected contact counts for different genotypes of the SNP rs11980001. The numbers on top are the 95th percentile values of the respective contact maps. **(G)** Same as (F) for the SNP rs6464142. **(H)** LD between all 6 connectivity iQTLs of the *NUB1* locus. **(I)** Genotype dependent variation of gene expression (eQTL trend) for the SNP rs11980001 and the gene *NUB1* in naïve CD8 cell type *(*adapted from the ImmuNexUT database). Boxplots show median (middle lines), 25th, 75th percentiles (box) and minimum and maximum range (whiskers). **(J)** Same trend as (I) with respect to the gene expression data of the DICE database for the naïve CD4 cell type. Boxplots indicate mean (bigger black dots), median (middle lines), 25th, 75th percentiles (box) and individual samples (smaller black dots). The p-value is obtained from linear regression coefficients (ANOVA). Obs: observed contact count, ExpCC: expected contact count. Source data are provided as a Source Data file.

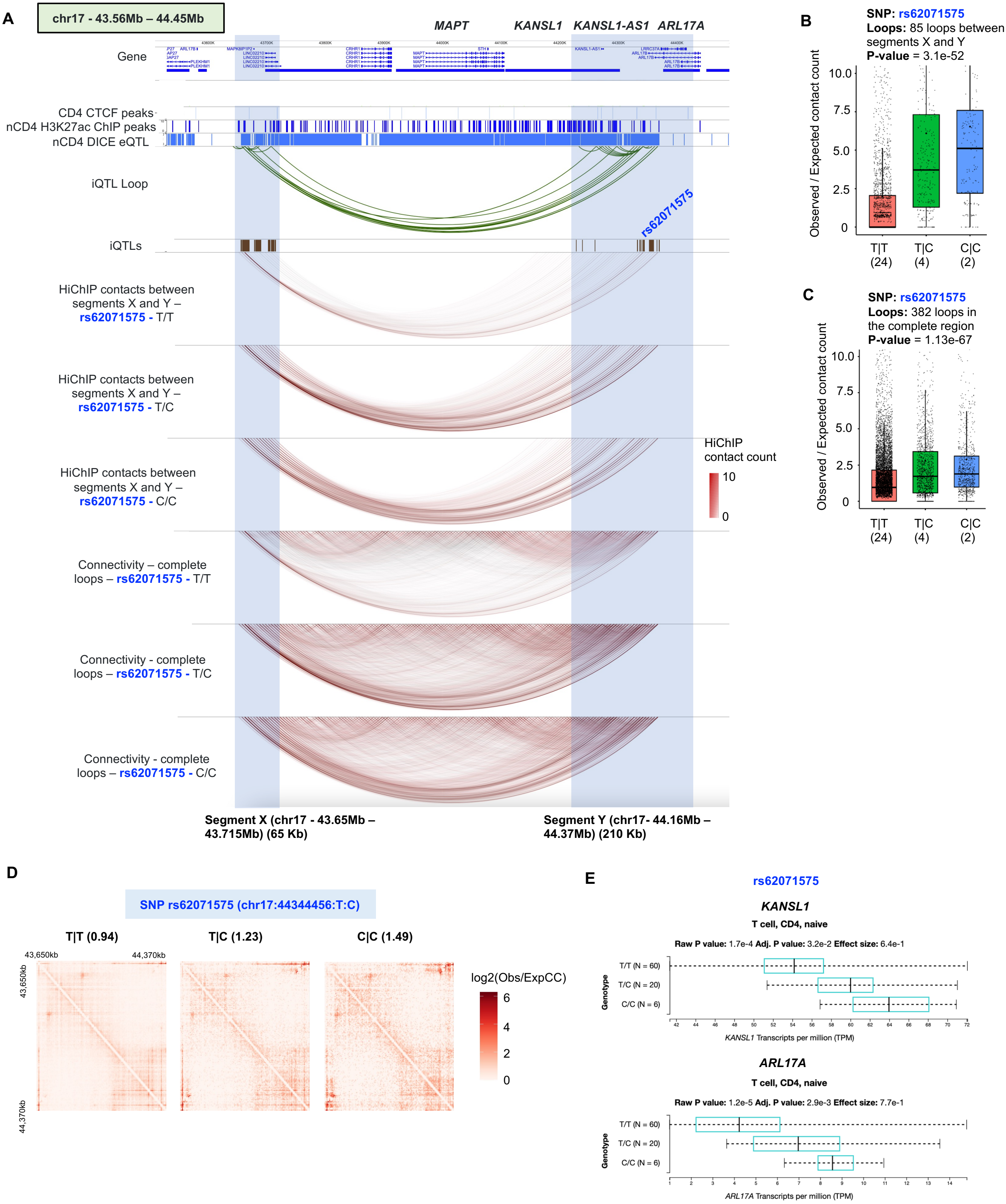

A
chr17 - 43.56Mb – 44.45Mb
MAPT
KANSL1
KANSL1-AS1
ARL17A
Gene
CD4 CTCF peaks
nCD4 H3K27ac ChIP peaks
nCD4 DICE eQTL
iQTL Loop
rs62071575
iQTLs
HiChIP contacts between segments X and Y – rs62071575 - T/T
HiChIP contacts between segments X and Y – rs62071575 - T/C
HiChIP contacts between segments X and Y – rs62071575 - C/C
HiChIP contact count
10
0
Connectivity – complete loops – rs62071575 - T/T
Connectivity - complete loops – rs62071575 - T/C
Connectivity - complete loops – rs62071575 - C/C
Segment X (chr17 - 43.65Mb – 43.715Mb) (65 Kb)
Segment Y (chr17- 44.16Mb – 44.37Mb) (210 Kb)
B
SNP: rs62071575
Loops: 85 loops between segments X and Y
P-value = 3.1e-52
Observed / Expected contact count
10.0
7.5
5.0
2.5
0
T|T (24)
T|C (4)
C|C (2)
C
SNP: rs62071575
Loops: 382 loops in the complete region
P-value = 1.13e-67
Observed / Expected contact count
10.0
7.5
5.0
2.5
0
T|T (24)
T|C (4)
C|C (2)
D
SNP rs62071575 (chr17:44344456:T:C)
T|T (0.94)
T|C (1.23)
C|C (1.49)
43,650kb
44,370kb
43,650kb
44,370kb
log2(Obs/ExpCC)
6
4
2
0
E
rs62071575
KANSL1
T cell, CD4, naive
Raw P value: 1.7e-4 Adj. P value: 3.2e-2 Effect size: 6.4e-1
T/T (N = 60)
T/C (N = 20)
C/C (N = 6)
Genotype
42
44
46
48
50
52
54
56
58
60
62
64
66
68
70
72
KANSL1 Transcripts per million (TPM)
ARL17A
T cell, CD4, naive
Raw P value: 1.2e-5 Adj. P value: 2.9e-3 Effect size: 7.7e-1
T/T (N = 60)
T/C (N = 20)
C/C (N = 6)
Genotype
1
2
3
4
5
6
7
8
9
10
11
12
13
14
ARL17A Transcripts per million (TPM)

Supplementary Fig. 10: Example of a common inversion polymorphism detected as a connectivity-QTLs in *KANSL1* locus. (A) rs62071575 is a perfect tagging SNP for this ~600kb chr17 inversion and is detected as a connectivity-QTL. Loops between the segments X and Y are used to determine the connectivity iQTLs. Three tracks below the list of iQTLs show the genotype-dependent changes of HiChIP contact counts for the loops between these segments. The bottom three tracks depict the genotype-dependent changes of HiChIP contact counts for the complete set of HiChIP loops in this locus. **(B)** Genotype-dependent trend of observed vs expected contact counts for the SNP rs62071575 and for all the loops between segments X and Y (indicated by the tracks *Connectivity between segments X and Y* in (A)). X axis indicates different genotypes and corresponding number of donors. Boxplots show median (middle lines), 25th, 75th percentiles (box) and individual samples (black dots). The p-value is obtained from linear regression coefficients (ANOVA). **(C)** Same as (B) but here the complete set of HiChIP loops in this locus (indicated by the tracks *Connectivity – complete loops* in (A)) are considered. **(D)** Aggregated contact map (log2-transformed observed vs expected contact counts) for different genotypes of the SNP rs62071575. The numbers on top are the 95th percentile values of the respective contact maps. **(E)** Genotype-dependent expression of the genes *KANSL1* (top) and *ARL17A* (bottom) for the SNP rs62071575 (adapted from DICE database). Boxplots show median (middle lines), 25th, 75th percentiles (box) and minimum and maximum range (whiskers). Obs: observed contact count, ExpCC: expected contact count. Source data are provided as a Source Data file.
