# Supplementary material for: Identifying genetic variants associated with chromatin looping and genome function
Source: Nat Commun. 2024 Sep 18;15:8174. doi: 10.1038/s41467-024-52296-4 (PMC11408621; doi:10.1038/s41467-024-52296-4)
Supplement: Supplementary file 3 — Description of Additional Supplementary Files [file 41467_2024_52296_MOESM3_ESM.pdf]

Description of Additional Supplementary Files

Supplementary Data 1: List of donors used in iQTL analysis (H3K27ac HiChIP, ChIP-seq and genotyping)
Supplementary Data 2: ChIP-seq peaks inferred by MACS2 on the merged ChIP alignment from all input donors.
Supplementary Data 3: Individual HiChIP samples, number of reads and the number of FitHiChIP significant interactions.
Supplementary Data 4: Complete list of iQTLs and their associated statistics
Supplementary Data 5: Complete list of DICE CD4 Naïve eQTLs (for autosomal chromosomes)
Supplementary Data 6: Overlap of iQTLs / eQTLs with fine mapped GWAS SNPs for various immune diseases
Supplementary Data 7: Stratified LD Score Regression (S-LDSC) based heritability enrichment for different categories of iQTLs and eQTLs
Supplementary Data 8: List of eGenes for which iQTLs are also their lead (most significant) eQTLs in CD4 Naive T cells, according to either DICE or ImmunexUT databases
Supplementary Data 9: List of iQTLs which are not eQTLs in CD4 Naïve (by DICE or ImmunexUT) but are eQTLs in different CD4 T cell subsets, and corresponding eGenes are differentially expressed (FDR < 0.05) and upregulated (logFC>1) in the respective CD4 T cell subset.
Supplementary Data 10: Complete list of Connectivity-QTLs and associated loci
Supplementary Data 11: Statistical significance for various overlap analysis
Supplementary Data 12: dbGAP HiChIP data repository for the iQTL related HiChIP samples
