# Supplementary material for: Identifying genetic variants associated with chromatin looping and genome function
Source: Nat Commun. 2024 Sep 18;15:8174. doi: 10.1038/s41467-024-52296-4 (PMC11408621; doi:10.1038/s41467-024-52296-4)
Supplement: Supplementary file 16 — Reporting Summary [file 41467_2024_52296_MOESM16_ESM.pdf]

Corresponding author(s): SOURYA BHATTACHARYYA (sourya@lji.org), FERHAT AY (ferhatay@lji.org)

Last updated by author(s): Aug 22, 2024

Reporting Summary

Nature Portfolio wishes to improve the reproducibility of the work that we publish. This form provides structure for consistency and transparency in reporting. For further information on Nature Portfolio policies, see our Editorial Policies and the Editorial Policy Checklist.

Statistics

For all statistical analyses, confirm that the following items are present in the figure legend, table legend, main text, or Methods section.

n/a	Confirmed	
☐	☒	The exact sample size (*n*) for each experimental group/condition, given as a discrete number and unit of measurement
☐	☒	A statement on whether measurements were taken from distinct samples or whether the same sample was measured repeatedly
☐	☒	The statistical test(s) used AND whether they are one- or two-sided<br>*Only common tests should be described solely by name; describe more complex techniques in the Methods section.*
☐	☒	A description of all covariates tested
☐	☒	A description of any assumptions or corrections, such as tests of normality and adjustment for multiple comparisons
☐	☒	A full description of the statistical parameters including central tendency (e.g. means) or other basic estimates (e.g. regression coefficient) AND variation (e.g. standard deviation) or associated estimates of uncertainty (e.g. confidence intervals)
☐	☒	For null hypothesis testing, the test statistic (e.g. *F*, *t*, *r*) with confidence intervals, effect sizes, degrees of freedom and *P* value noted<br>*Give P values as exact values whenever suitable.*
☒	☐	For Bayesian analysis, information on the choice of priors and Markov chain Monte Carlo settings
☒	☐	For hierarchical and complex designs, identification of the appropriate level for tests and full reporting of outcomes
☐	☒	Estimates of effect sizes (e.g. Cohen's *d*, Pearson's *r*), indicating how they were calculated

Our web collection on statistics for biologists contains articles on many of the points above.

Software and code

Policy information about availability of computer code

Data collection

Data collected from following public repositories:
DICE (http://dice-database.org),
database of Genotypes and Phenotypes (dbGaP),
ADASTRA (https://adastra.autosome.org/),
1000 Genomes Project consortium (https://www.internationalgenome.org/),
DICE (https://dice-database.org)
ImmuNexUT (https://www.immunexut.org/),
ChIP-Atlas (https://chip-atlas.org/),
ReMaP database (https://remap2022.univ-amu.fr/),
CausalDB (http://www.mulinlab.org/causaldb/browse.html),
LD-scores (https://alkesgroup.broadinstitute.org/LDSCORE/),
Open Targets Genetics (https://genetics.opentargets.org/),
Association to Function knowledge portal (https://a2f.hugeamp.org/),
LDlink (https://ldlink.nci.nih.gov/),
GWAS Catalog (https://www.ebi.ac.uk/gwas),
FIVEx eQTL browser (https://fivex.sph.umich.edu/),
C Origami (https://github.com/tanjimin/C.Origami),
GEO and SRA, using either web-based interfaces or UNIX command line tools.

We also downloaded CD4 T cell type .hic data from merged 54 Hi-C libraries, from the link: http://bartzabel.ls.manchester.ac.uk/orozcolab/SNP2Mechanism/hic/merged/

HiChIP sequencing data has been uploaded in dbGaP (https://www.ncbi.nlm.nih.gov/projects/gap/cgi-bin/molecular.cgi?study_id=phs001703.v5.p1).

We used RNA-seq and genotype data of DICE donors from dbGaP (accession number phs001703.v1.p1)

We used CD4 Naïve H3K27ac HiChIP dataset of 6 donors, as used in the publication Chandra et al. Nature Genetics 2021, from dbGaP (accession number phs001703.v3.p1).

We also created a web browser https://ay-lab-tools.lji.org/iQTL/ listing all the derived iQTLs, connectivity-QTLs, corresponding looping information, genotype and allele-specific trend plots, and the WashU browser tracks for individual SNP-loop pairs.

Data analysis

ChIPLine (https://github.com/ay-lab/ChIPLine)
Fine-mapping pipeline (https://github.com/ay-lab/finemap)
Colocalization pipeline (https://github.com/ay-lab/Colocalization)
Stratified LD Score Regression pipeline (https://github.com/ay-lab/S_LDSC_SNP)
Bowtie2 (http://bowtie-bio.sourceforge.net/bowtie2/index.shtml)
Picard (http://broadinstitute.github.io/picard/)
Samtools (http://samtools.sourceforge.net/)
MACS2 (https://github.com/macs3-project/MACS)
HiC-pro (https://github.com/nservant/HiC-Pro)
FitHiChIP (https://github.com/ay-lab/FitHiChIP)
snpQC (https://cgondro2.une.edu.au/snpQC.htm)
PLINK (https://zzz.bwh.harvard.edu/plink/dataman.shtml)
IMPUTE2 (http://mathgen.stats.ox.ac.uk/impute/impute_v2.html)
GATK (https://gatk.broadinstitute.org/hc/en-us)
RASQUAL (https://github.com/natsuhiko/rasqual)
Bedtools (https://bedtools.readthedocs.io/en/latest/)
HOMER (http://homer.ucsd.edu/homer/)
FIMO (https://meme-suite.org/meme/doc/fimo.html)
AME (https://meme-suite.org/meme/doc/ame.html)
Stratified LD score regression(S-LDSC) (https://github.com/bulik/ldsc)
GENOVA (https://github.com/robinweide/GENOVA)
WashU Epigenome browser (https://epigenomegateway.wustl.edu/)
deepTools (https://deeptools.readthedocs.io/en/develop/)
C Origami (https://github.com/tanjimin/C.Origami)
UCSC hgLiftOver (https://genome.ucsc.edu/cgi-bin/hgLiftOver)
Metascape (https://metascape.org/gp/index.html#/main/step1)
Custom scripts written in R (https://www.r-project.org/) version 4.1

For manuscripts utilizing custom algorithms or software that are central to the research but not yet described in published literature, software must be made available to editors and reviewers. We strongly encourage code deposition in a community repository (e.g. GitHub). See the Nature Portfolio guidelines for submitting code & software for further information.

Data

Policy information about availability of data

All manuscripts must include a data availability statement. This statement should provide the following information, where applicable:

- Accession codes, unique identifiers, or web links for publicly available datasets
- A description of any restrictions on data availability
- For clinical datasets or third party data, please ensure that the statement adheres to our policy

All downloaded data is available through public repositories such as DICE, database of Genotypes and Phenotypes (dbGaP), ADASTRA, 1000 Genomes Project consortium, ImmuNexUT, ChIP-Atlas, REMAP database, CausalDB, LD-scores, Open Targets Genetics, Association to Function knowledge portal, LDlink, GWAS Catalog, FIVEx eQTL browser, C. Origami, ENCODE, GEO and SRA.

The DICE project provides eQTL data for public access at http://dice-database.org. Individual–specific RNA-sequencing and genotype data are available from the database of Genotypes and Phenotypes (dbGaP Accession number: phs001703.v1.p1). HiChIP data of 6 donors (published in our earlier work Chandra et al. Nature Genetics 2021) are available from the database of Genotypes and Phenotypes (dbGaP Accession number: phs001703.v3.p1).

HiChIP sequencing data has been uploaded in dbGAP (https://www.ncbi.nlm.nih.gov/projects/gap/cgi-bin/molecular.cgi?study_id=phs001703.v5.p1).

We also created a web browser https://ay-lab-tools.lji.org/iQTL/ listing all the derived iQTLs, connectivity-QTLs, corresponding looping information, genotype and allele-specific trend plots, and the WashU browser tracks for individual SNP-loop pairs.

We also uploaded the results corresponding to the manuscript in Zenodo repository (10.5281/zenodo.13127086).

We also uploaded the source code and data analysis scripts in GitHub as open source repositories.
1. iQTL pipeline and associated scripts is hosted at https://github.com/ay-lab/iQTL
2. Fine mapping pipeline is hosted at https://github.com/ay-lab/finemap
3. Colocalization script is provided at https://github.com/ay-lab/Colocalization
4. Script for Stratified LD Score Regression (S-LDSC) applied on SNPs is provided at https://github.com/ay-lab/S_LDSC_SNP

The ChIP-seq peaks, HiChIP loops for all the samples, and the list of IQTLs and connectivity-QTLs are shared as part of the supplementary tables of this manuscript.

Research involving human participants, their data, or biological material

Policy information about studies with human participants or human data. See also policy information about sex, gender (identity/presentation), and sexual orientation and race, ethnicity and racism.

Reporting on sex and gender	We reported the sex information for individual donors in the supplementary table 1. We used this sex information as a covariate in our method to identify the significant QTLs (described in the manuscript and also in the METHODS section).
Reporting on race, ethnicity, or other socially relevant groupings	We reported the ethnicity and race information for individual donors in the supplementary table 1. We used this information as a covariate in our method to identify the significant QTLs (described in the manuscript and also in the METHODS section).
Population characteristics	We reported the age information for individual donors in the supplementary table 1. We used this information as a covariate in our method to identify the significant QTLs (described in the manuscript and also in the METHODS section).
Recruitment	The samples were part of the DICE study (https://dice-database.org)
Ethics oversight	NA

Note that full information on the approval of the study protocol must also be provided in the manuscript.

Field-specific reporting

Please select the one below that is the best fit for your research. If you are not sure, read the appropriate sections before making your selection.

☒ Life sciences ☐ Behavioural & social sciences ☐ Ecological, evolutionary & environmental sciences

For a reference copy of the document with all sections, see nature.com/documents/nr-reporting-summary-flat.pdf

Life sciences study design

All studies must disclose on these points even when the disclosure is negative.

Sample size	30 samples were used to generate the HiChIP datasets. The sample size is indicated in the figure legends.
Data exclusions	No data is excluded.
Replication	HiChIP was performed on 30 biological replicates.
Randomization	Randomization was not performed. The employed methods involve unbiased quantification. Hence, the data presented did not require randomization.
Blinding	Blinding was not performed. The employed methods involve unbiased quantification. Hence, the data presented did not require blinding.

Reporting for specific materials, systems and methods

We require information from authors about some types of materials, experimental systems and methods used in many studies. Here, indicate whether each material, system or method listed is relevant to your study. If you are not sure if a list item applies to your research, read the appropriate section before selecting a response.

Materials & experimental systems

n/a	Involved in the study	
☐	☒	Antibodies
☒	☐	Eukaryotic cell lines
☒	☐	Palaeontology and archaeology
☒	☐	Animals and other organisms
☒	☐	Clinical data
☒	☐	Dual use research of concern
☒	☐	Plants

Methods

n/a	Involved in the study	
☐	☒	ChIP-seq
☐	☒	Flow cytometry
☒	☐	MRI-based neuroimaging

Antibodies

Antibodies used	Premium H3K27ac polyclonal antibody from Diagenode (C15410196) was used for HiChIP.

Validation	Antibody has been validated by the respective manufacturer for flow cytometry

Plants

Seed stocks	NA
Novel plant genotypes	NA
Authentication	NA

ChIP-seq

Data deposition

☐ Confirm that both raw and final processed data have been deposited in a public database such as GEO.

☒ Confirm that you have deposited or provided access to graph files (e.g. BED files) for the called peaks.

Data access links *May remain private before publication.*	Supplementary Table 2 of this study contains the ChIP-seq peaks.
Files in database submission	*Provide a list of all files available in the database submission.*
Genome browser session (e.g. UCSC)	WashU browser session ID containing the interaction QTLs, HiChIP loop calls, and the ChIP-seq peaks: c730ec60-4de0-11ef-8802-7f6b1b69f09b<br><br>User needs to go to https://epigenomegateway.wustl.edu/, click "load a session", provide this session ID, click "retrieve", select "Restore" along "iQTL_Sourya_July2024" to visualize this tracks.<br><br>We also created a web browser https://ay-lab-tools.lji.org/iQTL/ listing all the derived iQTLs, connectivity-QTLs, corresponding looping information, genotype and allele-specific trend plots, and the WashU browser tracks for individual SNP-loop pairs.

Methodology

Replicates	30 biological replicates were used to provide sufficient confidence to conclusions derived from primary cell H3K27ac HiChIP.
Sequencing depth	Libraries were sequenced on an Illumina HiSeq 2500 sequencer to obtain 50-bp single-end reads. Details of individual study subjects are provided in Supplementary Table 1.
Antibodies	Premium H3K27ac polyclonal antibody from Diagenode (C15410196) was used for HiChIP.
Peak calling parameters	Processing of ChIP-seq peaks and HiChIP data, and generating the HiChIP loop calls, are described in the supplementary METHODS section.
Data quality	Number of ChIP-seq peaks with FDR < 5% and fold change >= 5 are: 12688. Number of HiChIP loops and sequencing depth (number of valid CIS read pairs) for individual donors are indicated in the Supplementary Table 3.
Software	ChIP-seq data processing as mentioned above, was done with a custom pipeline named ChIPLine developed in our lab (https://github.com/ay-lab/ChIPLine). HiChIP data loop calling was done using our previously published method FitHiChIP (https://github.com/ay-lab/FitHiChIP)

Flow Cytometry

Plots

Confirm that:

- [x] The axis labels state the marker and fluorochrome used (e.g. CD4-FITC).
- [x] The axis scales are clearly visible. Include numbers along axes only for bottom left plot of group (a 'group' is an analysis of identical markers).
- [x] All plots are contour plots with outliers or pseudocolor plots.
- [x] A numerical value for number of cells or percentage (with statistics) is provided.

Methodology

Sample preparation	Peripheral blood mononuclear cells (PBMC) were obtained from leukapheresis samples by density gradient centrifugation and cryopreserved in liquid nitrogen. For the isolation of immune cell types of interest, cryopreserved PBMCs were thawed, washed, stained directly with cocktails of fluorescently conjugated antibodies or pre-enriched for total B cells using the 'Human B Cell Isolation Kit II' (Miltenyi Biotec), following the manufacturer's instructions before staining with antibodies and sorted on a BD FACSAria II (Becton Dickinson) using the gating strategies as described (Schmiedel et al., 2018, Cell).
Instrument	BD LSRFortessa
Software	BD FACSDiva<br>FlowJo v10.4.1
Cell population abundance	Sorting efficiency was observed during sorting and didn't drop below 90%.
Gating strategy	Gating strategy as mentioned in Schmiedel et al., 2018, Cell.

- [ ] Tick this box to confirm that a figure exemplifying the gating strategy is provided in the Supplementary Information.
